# Enhancing Signal and Network Integrity: Evaluating BCG Artifact Removal Techniques in Simultaneous EEG-fMRI Data

**DOI:** 10.3390/s25227036

**Published:** 2025-11-18

**Authors:** Perihan Gülşah Gülhan, Güzin Özmen

**Affiliations:** 1 Department of Electrical Electronics Engineering, Institute of Science, Selcuk University, Konya 42130, Turkey; perihangulsahgulhan@ankara.edu.tr; 2 Department of Optoelectronics and Electro-Optic Technologies, Akyurt Vocational School, Ankara University, Ankara 06935, Turkey; 3 Department of Biomedical Engineering, Selcuk University, Konya 42075, Turkey

**Keywords:** BCG artifact removal, brain graph metrics, multimodal data analysis, simultaneous EEG-fMRI, signal quality assessment, functional connectivity

## Abstract

**Highlights:**

In this study, we systematically investigated the effects of different BCG artifact removal methods on EEG signal quality and functional connectivity during simultaneous EEG–fMRI recordings. The analysis demonstrated how various methods impact signal fidelity, structural similarity, and dynamic graph metrics across EEG frequency bands. The results revealed method-specific differences in network topology, highlighting the importance of choosing an appropriate artifact removal approach for accurate brain network interpretation.

**What are the main findings?**

- AAS achieved the best signal fidelity (MSE = 0.0038, PSNR = 26.34 dB), and OBS yielded the highest structural similarity (SSIM = 0.72), whereas ICA showed greater sensitivity in dynamic graph metrics.
- Connectivity graphs after artifact removal displayed distinct frequency-specific patterns, particularly in the beta and gamma bands.

**What are the implications of the main findings?**

- Artifact removal affects both EEG signal preprocessing and the topological interpretation of the functional brain networks.
- Method selection is critical for reliable connectivity and graph-based analyses in EEG–fMRI studies.

**Abstract:**

Simultaneous Electroencephalography (EEG) and functional Magnetic Resonance Imaging (fMRI) provide a powerful framework for investigating brain dynamics; however, ballistocardiogram (BCG) artifacts in EEG compromise signal quality and limit the assessment of brain connectivity. This study evaluated three widely used artifact removal methods—Average Artifact Subtraction (AAS), Optimal Basis Set (OBS), and Independent Component Analysis (ICA)—together with two hybrid approaches (AAS + ICA and OBS + ICA). Unlike previous studies that focused solely on signal-level metrics, we adopted a holistic framework that combined signal quality indicators with graph-theoretical analysis of EEG-fMRI connectivity in static and dynamic contexts. The results show that AAS provides the best signal quality, whereas OBS better preserves structural similarity. ICA, although weaker in terms of signal metrics, demonstrates sensitivity to frequency-specific patterns in dynamic graphs. Hybrid methods yield benefits, with OBS + ICA producing the lowest *p*-values across frequency band pairs (e.g., theta–beta and delta–gamma), particularly in dynamic graphs. Topological analyses revealed that artifact removal significantly affected network structure, with dynamic analyses showing more pronounced frequency-specific effects than static analyses. High-frequency bands, such as beta and gamma, exhibit stronger differentiation under dynamic conditions. Overall, this study offers new insights into the relationship between artifact removal and brain network integrity, emphasizing the need for multimodal and frequency-sensitive evaluation strategies. The findings guide preprocessing decisions in EEG-fMRI studies and clarify how methodological choices shape the interpretation of brain connectivity.

## 1. Introduction

A comprehensive understanding of brain function requires methods that can simultaneously capture rapid neural activity and map the interactions across brain regions. Electroencephalography (EEG) offers millisecond-level precision in recording electrical activity [1], whereas functional Magnetic Resonance Imaging (fMRI) provides detailed maps of hemodynamic changes between brain regions [2]. Instead of treating each modality separately, multimodal fusion takes advantage of complementary features to enhance the overall analysis [3]. The integration of simultaneously collected data allows for a comprehensive examination of brain dynamics across different spatial and temporal scales [4,5]. However, the magnetic field generated by the MRI device during simultaneous recording introduces artifacts in the EEG data and significant signal distortion [6]. EEG signals are susceptible to artifacts that obscure true brain signals, such as muscle activity, eye movements, and heartbeat [7]. Ballistocardiogram (BCG) artifacts, particularly those resulting from the sensitivity of EEG electrodes to cardiac motion, complicate the simultaneous use of both methods [8]. Various preprocessing and analysis steps are required to extract meaningful insights from EEG and fMRI data. Brain graphs derived from these analyses play a crucial role in revealing the patterns of functional connectivity and temporal dynamics. However, the reliability of these findings depends critically on the effective removal of artifacts during preprocessing. Previous studies have predominantly assessed BCG artifact removal methods based on their performance in cleaning signal-level data. However, it is important to recognize that such artifacts not only degrade signal quality but also distort the functional connectivity structures derived from EEG-fMRI data, potentially biasing neuroscientific interpretations. To address the limitations of prior studies that focused solely on signal-level evaluations, this study makes the following contributions.

- Artifact removal methods (Average Artifact Subtraction (AAS), Optimal Basis Set (OBS), and Independent Component Analysis (ICA)) were evaluated not only in terms of signal quality but also in terms of their topological effects on functional connectivity.
- The signal-level performance was analyzed in a multifaceted manner using independent metrics, such as the Mean Squared Error (MSE), Peak Signal-to-Noise Ratio (PSNR), Signal-to-Noise Ratio (SNR), Structural Similarity Index (SSIM), Dynamic Time Warping (DTW), and Peak-to-Peak Ratio (PPR). The preservation of the frequency components was examined using Power Spectral Density (PSD) analysis. The EEG and fMRI time series were combined on a correlation basis, and static and dynamic brain plots were generated for each frequency band.
- Graph theory metrics, such as Connection Strength (CS), Clustering Coefficient (CC), and Global Efficiency (GE), were used to systematically analyze the effects of the methods on functional connectivity patterns.

This study introduces a comprehensive and innovative approach that considers artifact removal not only as a preprocessing step but also as a critical factor influencing the reliability of connectivity-based brain analysis.

### Related Works

The simultaneous use of EEG and fMRI enables the investigation of brain activity with both high temporal and spatial resolutions. However, EEG signals recorded during fMRI are often distorted by BCG artifacts caused by head movements that are synchronized with the cardiac cycle. These artifacts degrade the signal quality and may lead to inaccurate neural interpretations. Therefore, effective BCG artifact removal is critical for reliable EEG-fMRI studies to be conducted. This section reviews signal processing and hardware-based approaches for BCG artifact reduction and explores methods for integrating EEG and fMRI data to analyze functional brain networks.

A recent study used a combined EEG-fMRI approach to investigate the temporal and spatial neural dynamics involved in processing task-irrelevant emotional facial stimuli in patients with schizophrenia [9]. This study examined abnormalities in facial recognition across both modalities, highlighting the importance of multimodal analysis in psychiatric disorders.

Numerous approaches have been proposed for removing BCG artifacts from simultaneous EEG-fMRI recordings. The earliest studies began with templates [10] that introduced the AAS method. AAS, a template-based approach, aims to identify BCG artifacts in EEG data during fMRI and remove them by averaging the templates. Although AAS is an easy-to-apply method, it cannot adequately represent variations in artifacts across individuals and over time. The OBS method was later proposed to better capture the temporal and structural variability of BCG artifacts. In the same study, a FASTR algorithm was introduced to automate the OBS. OBS uses Principal Component Analysis (PCA) to identify and extract dominant variations in artifact structures [11]. However, the effectiveness of the OBS method may vary depending on the dataset and channel, and better results can be obtained with adaptive versions of this method [12]. ICA-based approaches can also remove artifacts from EEG data by decomposing them into different components [13,14]. The effectiveness of this method depends on the correct identification of the components [15]. However, component selection requires expertise, and the removal of an incorrect component can lead to the loss of neural information. Many ICA algorithms and different component selection methods have been used to remove BCG artifacts [16]. ICA can be implemented using various strategies, including linear regression or hybrid combinations. In one study, joint independent component analysis (jICA) was used to measure the relationship between EEG and fMRI signals [17]. Joint Independent Component Analysis (JICA) is a Blind Source Separation (BSS) approach for combining and analyzing data obtained from different neuroimaging modalities [18]. It is an extension of the classical ICA but attempts to analyze multiple datasets simultaneously.

The most successful algorithms for removing BCG artifacts are Infomax [14,19] and Fast ICA [13]. In one study, the Low-Rank + Sparse Decomposition (LR + SD) algorithm was proposed as an alternative to the applied methods [20]. The LR + SD algorithm is based on the assumption that data events are sparsely represented in the time domain and that artifacts propagate between the channels on the head surface. In this study, the LR + SD method was compared with ICA, and the SNR was significantly improved [20].

Levitt et al. (2023) introduced EEG-LLAMAS, an open-source, low-latency software platform for real-time BCG artifact removal [21]. Through simulations based on steady-state visually evoked potentials (SSVEPs) and real-time EEG-fMRI experiments, they demonstrated that LLAMAS outperformed traditional methods, such as OBS, in recovering EEG signals and power spectra with an average latency of less than 50 ms. This makes it particularly valuable for closed-loop EEG-fMRI paradigms.

In addition to the AAS, OBS, and ICA, some studies have used a combination of these methods to remove BCG artifacts [15,22,23]. In a study using the same dataset, the combination of OBS-ICA improved the artifact removal results. The effectiveness of these methods can vary depending on the choice of parameters used and the optimization processes. However, there is no clear superiority among existing methods, thus emphasizing the need for hardware-based solutions [23].

Hardware-based approaches include the integration of piezoelectric sensors or extra electrodes into the EEG head. These tools have been used to generate reference signals and reduce BCG artifacts [24,25]. Innovative hardware solutions have also been proposed, such as camera-based motion correction [26] and the use of carbon-fiber wire suspension [27]. However, these approaches often have limitations such as high cost, complex setup, and long lead times [28].

In recent years, analysis of functional connectivity using graph theory has gained popularity in neuroscience. This mathematical framework models brain networks using nodes and edges to describe interregional connectivity [29]. Functional connectivity derived from EEG and fMRI is typically visualized using static or dynamic brain graphs. For example, ref. [30] investigated EEG-fMRI connectivity patterns using graph metrics, and examined how they varied across frequency bands.

Beyond graph-theoretical approaches, recent developments in multimodal data fusion techniques have enabled more precise extraction of shared and distinct brain activity patterns from EEG and fMRI recordings. One such method is the Generalized Coupled Matrix Tensor Factorization (GCMTF) model, which utilizes normalized mutual information (NMI) to capture both linear and nonlinear dependencies between modalities. This approach improves upon the traditional ACMTF framework by relaxing the restrictive assumption of component equality in shared dimensions. Application of the GCMTF method to simulated and real auditory oddball data has demonstrated significantly enhanced component matching accuracy and identification of functionally relevant brain regions, particularly in the alpha and theta bands [31].

Recent studies have aimed to uncover the relationship between temporal EEG spectral dynamics and spatial dynamic functional brain networks obtained using resting-state fMRI. One of these studies estimated voxel-based dynamic brain networks and examined their temporal relationships with simultaneously recorded EEG spectral bands [32]. By calculating time-varying connectivity matrices and performing cross-correlation analyses, this study identified specific network pairs whose spatial dynamics were significantly correlated with fluctuations in resting-state EEG spectral power. This multimodal integration provides valuable insights into the complex spatiotemporal structure of the brain.

Another study presented a novel approach to correlate voxel-based dynamic fMRI networks derived using spatially constrained ICA with time-varying EEG spectral power across the delta, theta, alpha, and beta bands. The results revealed significant correlations, including increased alpha power associated with greater volume in the primary visual network during rest with eyes open, and motor network dynamics consistent with alpha and beta band modulations. These findings demonstrate the importance of the complementary strengths of EEG and fMRI in capturing the spatiotemporal structure of the brain [33].

Most existing studies have evaluated BCG artifact removal methods, primarily based on signal-level improvements. However, artifacts may also distort derived brain connectivity structures and influence the interpretation of neural processes. To bridge this gap, the present study systematically evaluates AAS, OBS, ICA, and their hybrid combinations using multiple signal-quality metrics (MSE, PSNR, SNR, SSIM, DTW, and PPR) that assess signal similarity, structural preservation, timing coherence, and frequency integrity. PSD analysis was also used to visually assess the spectral conservation. Moreover, EEG and fMRI time series were integrated to construct static and dynamic brain connectivity graphs across the five frequency bands. The resulting graphs were analyzed using topological metrics (CS, CC, and GE) to quantify the impact of artifact removal techniques on the brain connectivity patterns. In this respect, this study is one of the first to systematically evaluate the topological effects of BCG artifact removal methods on connectivity in addition to their signal-level performance.

## 2. Materials and Methods

**Figure 1:**
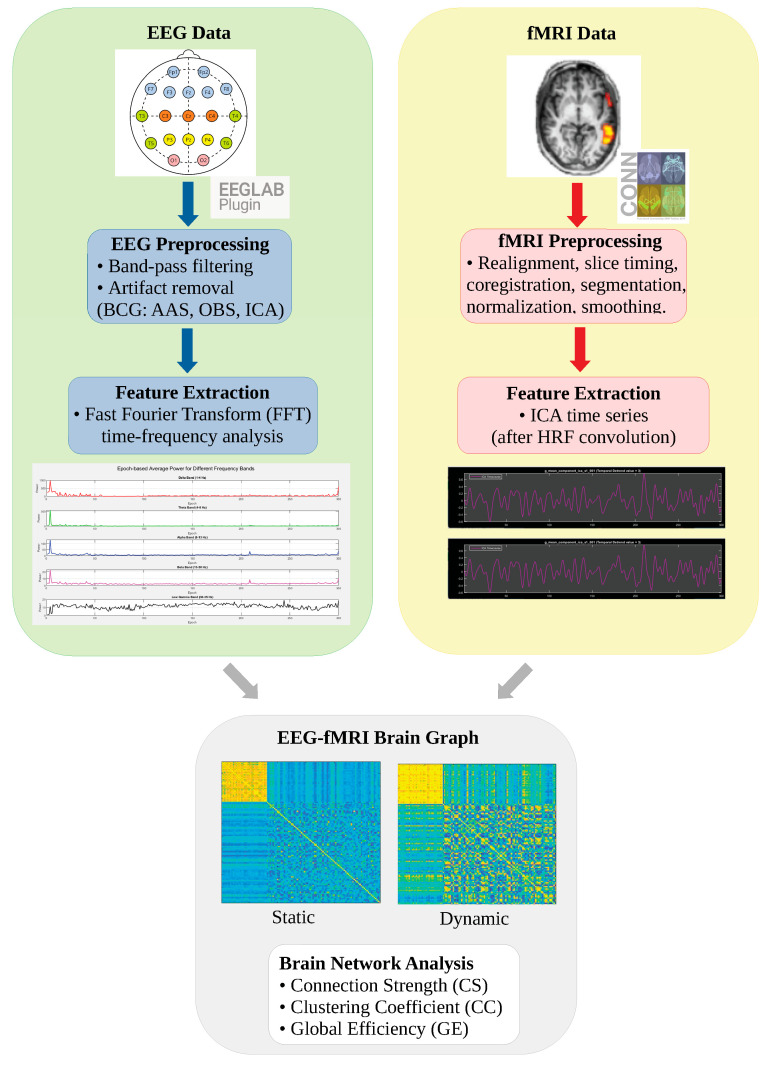
Schematic Overview of the Study Workflow. Color coding: Green indicates EEG processing pipeline; Yellow indicates fMRI processing pipeline; Gray represents the integrated EEG-fMRI brain graph analysis.

This section describes the procedures for EEG and fMRI data acquisition, preprocessing, and analysis as well as the methods used to construct EEG-fMRI brain graphs. In addition, the metrics employed to assess the impact of the three different artifact removal techniques on EEG signal quality are explained in detail. An overview of the workflow of this study is shown in Figure 1.

### 2.1. Dataset

This study utilized data from the publicly available “Simultaneous EEG-fMRI Dataset” hosted on the Mendeley Data Repository [34]. The dataset included simultaneous EEG and fMRI recordings from 20 healthy male participants (mean age = 26 years, standard deviation = 3.8 years) and was structured according to the Brain Imaging Data Structure (BIDS) standard. After preliminary data quality checks and outlier analysis, data from 5 participants were excluded due to excessive artifacts or missing data, resulting in a final sample of 15 participants.

EEG recordings were acquired using an MR-compatible Geodesic EEG System 400 (GES 400 MR) with a 32-channel sponge-based Geodesic Sensor Net, referenced to Cz and sampled at 1000 Hz. The dataset provided EEG data recorded under three distinct conditions.

- Outside the MRI scanner (artifact-free EEG),
- Inside the scanner without fMRI acquisition (EEG affected only by BCG artifacts),
- During simultaneous EEG-fMRI acquisition (EEG was affected by both the MRI gradient and BCG artifacts).

Although the original dataset includes both resting-state and task-based (EOEC: Eyes Open–Eyes Closed transition) EEG recordings, only the resting-state data were analyzed in this study to ensure a controlled evaluation of BCG artifact removal techniques without task-related neural confounds. The decision to focus exclusively on resting-state data was made to minimize variability introduced by cognitive or motor processes during tasks, enabling a more direct comparison of artifact correction performance. fMRI data were acquired using Echo Planar Imaging (EPI) sequences on a 3 Tesla GE Discovery MR750 scanner with a 32-channel head coil. The imaging parameters were as follows: field of view (FOV) = 25.6 cm, matrix size = 64 × 64, repetition time (TR) = 2000 ms, echo time (TE) = 40 ms, number of slices = 35, voxel size = 4 mm^3^. EEG data were stored in .set and .fdt format, whereas fMRI data were stored in .nii (NIfTI) format. The fMRI recordings included resting state and task-based data. Consistent with the EEG data selection, only the resting-state fMRI recordings were used in the present study. This ensured methodological consistency between EEG and fMRI modalities and allowed the evaluation of artifact removal performance under stable neural conditions.

### 2.2. EEG Preprocessing

The EEG data from all participants were preprocessed using the EEGLAB toolbox (v2024.1) [35] implemented in MATLAB (R2021a; MathWorks, Natick, MA, USA). First, a 1–50 Hz band-pass filter was applied to eliminate both high-frequency noise and low-frequency drifts from the signal.

#### 2.2.1. BCG Artifact Removal Methods

BCG artifacts were removed by detecting QRS complexes in the Electrocardiogram (ECG) channel (Channel 33) and using them as temporal reference points. FMRIB plugin integrated into EEGLAB was used, which provides high sensitivity and specificity for QRS detection. Three commonly used subtraction methods, namely, AAS, OBS, and ICA, were then independently applied to the EEG data for each participant. In the AAS method, an artifact template is generated by centering these segments, and a clean signal is obtained by subtracting the template from each segment. The OBS method employs a PCA-based approach to better account for the temporal variability of artifacts. In this study, both AAS and OBS were implemented using the FASTR plugin [11] via the fMRIB toolbox integrated into EEGLAB. The EEG signal was segmented into QRS-triggered epochs based on cardiac events detected from the ECG channel of each participant. In the AAS method, an artifact template is generated by centering these segments, and the cleaned signal is subtracted from each segment. In this study, the average artifact pattern was calculated using the AAS method over segments corresponding to 30 heart beats. For this calculation, the low-pass filter cutoff frequency was set to 70 Hz and the interpolation factor was set to 10. In the OBS method, an artifact matrix is constructed from these segments, and PCA is applied. The PCA component selection for the OBS was set to the default value of *N* = 4 Principal Components (PCs). This basis set was subsequently fitted and subtracted from each segment to minimize residual BCG artifacts.

**Figure 2:**
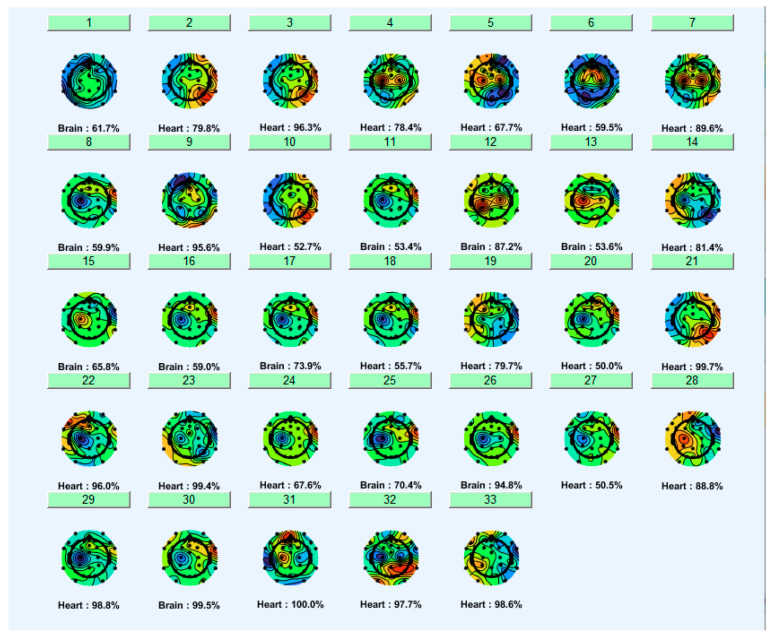
Topographic maps of independent components labeled following ICA decomposition. Each map shows the spatial distribution of an independent component on the scalp. Black dots represent electrode positions. Green indicates no component effect, red indicates positive contributions, and blue indicates negative contributions. Below each component is its estimated source and the percentage similarity (%) to that source.

The ICA method decomposes EEG signals into statistically independent components (ICs). In this study, the Runica algorithm in EEGLAB was used to perform the ICA. The EEG signal was decomposed into ICs, each of which was analyzed based on its temporal profile and topographic map. Independent Component Labeling (ICLabel) [36], an automated EEG IC classification plugin, was used to identify components. Figure 2 displays the components labeled by ICLabel, distinguishing between brain activity and various types of artifacts. ICLabel categorizes ICA components into classes, such as brain activity, eye movements, cardiac activity, muscle artifacts, line noise, and other sources. Components were automatically removed based on a strict probability threshold to ensure artifacts excision. Components labeled as ‘Heart’ and ‘Eye’ were automatically removed only if they had a classification probability threshold of 0.80 or greater. Components classified as ‘Brain’ were retained. This threshold-based approach enabled effective removal of components related to cardiac and ocular artifacts in the ICA.

As these three methods alone were insufficient for the removal of BCG artifacts, two hybrid methods, AAS + ICA and OBS + ICA, were also evaluated. In both hybrid methods, QRS detection was first performed, and the data were divided into epochs. In the AAS + ICA method, the AAS method was first applied to epoched data, followed by ICA-based artifact removal. In the OBS + ICA method, OBS was initially applied to the epoched data to remove high-variance artifact components using PCA, after with ICA was performed.

After artifact removal, the data were normalized to ensure comparability between individuals. The EEG signals were then divided into 2 s epochs and a total of 300 epochs were obtained for each participant. In the final stage of pre-processing, the ECG channel, which was not used in the analysis, was removed from the dataset.

#### 2.2.2. Performance Evaluation Metrics

This study employed several performance metrics to evaluate the quality of EEG signals after BCG artifact removal. Each metric was selected to capture the distinct characteristics of the signal, including the amplitude, structural similarity, temporal coherence, and frequency content, as summarized in Table 1. After artifact removal, a signal normalization step was performed to ensure comparability across participants and methods. Each EEG signal was normalized by dividing it by its maximum absolute amplitude value, bringing all data into a standardized range between −1 and +1. This normalization procedure prevents amplitude differences from influencing subsequent quantitative evaluations (e.g., MSE, PSNR, SSIM) and allows an objective comparison of the performance of the artifact removal methods.

For DTW, temporal similarity was computed using 1 s segments from both the raw and artifact-corrected signals. This short, fixed-length segment captures representative features of the signal while keeping the metric comparable across channels and artifact removal methods. Signals were already normalized prior to DTW computation, ensuring scale-independence of the DTW metric.

These metrics are used to evaluate not only the effectiveness of artifact removal methods in reducing noise but also their ability to preserve the structural, temporal, and spectral integrity of the signal.

### 2.3. EEG Analysis

Analyzing the spectral power of EEG signals is a common approach for studying electrical activity in the brain [33]. In this context, the effects of BCG artifacts on the frequency structure of EEG signals were evaluated by transforming the time-domain EEG data into the frequency domain using Fast Fourier Transform (FFT). Using this transformation, the power distribution of the signal in different frequency bands was analyzed. Within the scope of the analysis, five classical EEG frequency bands were used: Delta (1–4 Hz), Theta (4–8 Hz), Alpha (8–12 Hz), Beta (13–30 Hz), and Gamma (30–35 Hz). Each EEG frequency band corresponds to specific brain functions and cognitive activities [43,44]. Power spectra were calculated for each frequency band, and spectral power time series of [300 epochs × 32 channels] were obtained for each participant. Thus, the effects of artifact removal methods were comparatively evaluated in terms of frequency content, not only in the time domain.

**Figure 3:**
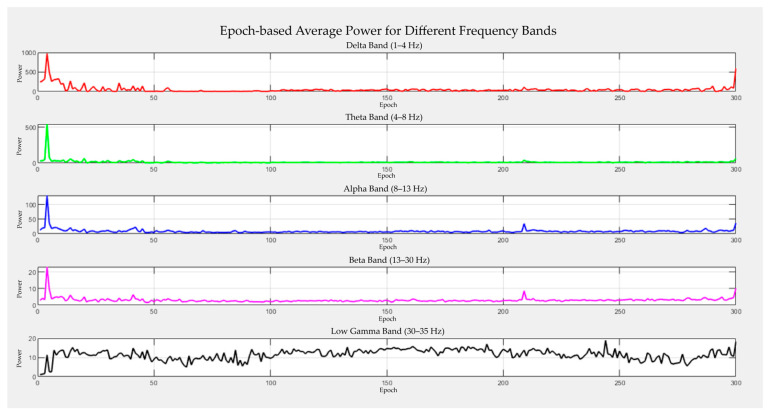
Average power spectra of a participant’s EEG data by frequency bands: Delta, Theta, Alpha, Beta and Gamma. The color coding for the power spectra lines is as follows: Delta Band (1–4 Hz) in Red, Theta Band (4–8 Hz) in Green, Alpha Band (8–13 Hz) in Blue, Beta Band (13–30 Hz) in Magenta (Pink), and Low Gamma Band (30–35 Hz) in Black.

Figure 3 shows the power distribution of the representative EEG signals across individual frequency bands. This analysis enables the assessment of the frequency-level effects of BCG artifacts and provides insights into how well artifact removal methods preserve the spectral integrity.

### 2.4. fMRI Preprocessing

fMRI data were pre-processed using the CONN toolbox (22. v2407) [45]. The preprocessing pipeline includes realignment, slice timing correction, co-registration, segmentation, normalization, and spatial smoothing. During the smoothing step, a Gaussian kernel of 5 mm Full Width at Half Maximum (FWHM) was applied to reduce the spatial noise and enhance the signal-to-noise ratio. Following preprocessing, a denoising procedure was applied to all participants’ fMRI data to remove artifacts caused by head motion, low-quality acquisition, and environmental noise. Additionally, band-pass filtering (0.01–0.08 Hz) was performed to retain low-frequency fluctuations associated with brain functional connectivity, while excluding irrelevant frequency components.

### 2.5. fMRI Analysis

Group-level fMRI analysis was performed using the Group ICA of fMRI Toolbox (GIFT) (v4.0.5.10) [46]. Initially, the dimensionality of each participant’s fMRI data was reduced using PCA and the 120 most significant components were retained per subject. The reduced data were concatenated across participants and ICA was performed using the Infomax algorithm [47] to extract 100 ICs.

**Figure 4:**
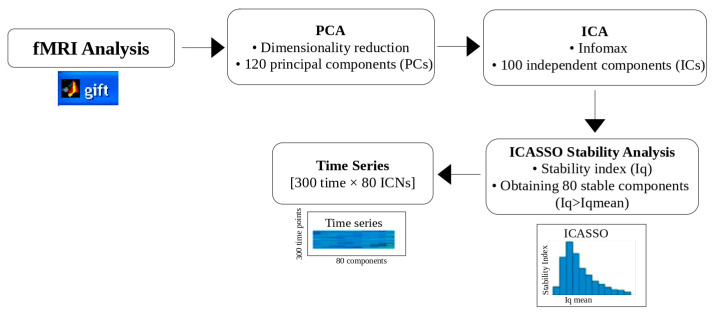
Workflow of the fMRI analysis process.

The reliability of the ICs, which perform 10 iterations of the ICA algorithm, was assessed using the ICASSO tool [48]. For each component, a stability index (Iq) ranging from 0 to 1. Higher Iq values indicate greater consistency across the iterations. Components with Iq values above the group mean were considered reliable, resulting in the selection of 80 ICs values for subsequent analyses. In the final step, time-series matrices for these 80 components were extracted for each participant, resulting in matrices of size [300-time points × 80 components]. Figure 4 illustrates the analysis pipeline, including the PCA-based dimensionality reduction, ICA decomposition, IC reliability assessment using ICASSO, and time series extraction.

**Figure 5:**
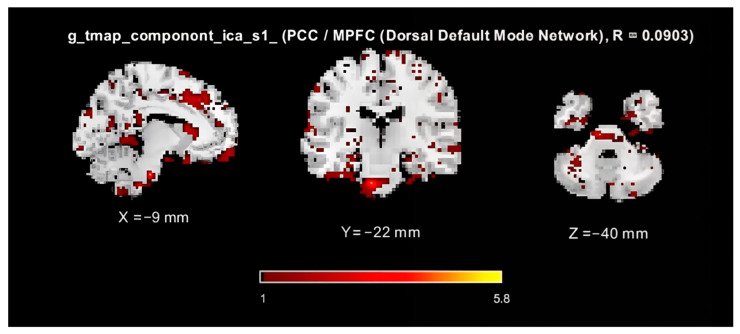
Spatial map of an independent component corresponding to the DMN.

In the fMRI analysis, each component obtained through ICA was analyzed based on its temporal dynamics and spatial distribution. Figure 5 presents a spatial map of an independent component corresponding to the Default Mode Network (DMN). This map visualizes the spatial extent and activation patterns of components across different brain regions. The DMN is a well-known network associated with self-referential thinking and mind-wandering, and is typically active during rest. The spatial representation of this component clearly demonstrates the brain regions involved and the organizational structure of the network used in the analysis. Figure 5 illustrates the spatial distribution of brain activity associated with an ICA-derived component that overlaps the DMN, highlighting the brain regions engaged during resting-state processes.

### 2.6. EEG-fMRI Brain Graph Construction

This section details the integration of multimodal information from simultaneous EEG-fMRI recordings to construct brain graphs. In the earlier stages of the study, EEG signals were denoised, temporal features were extracted across specific frequency bands, and fMRI data underwent spatial normalization and pre-processing. These datasets were then temporally and spatially aligned, making them suitable for subsequent correlation-based connectivity analysis.

#### 2.6.1. Convolution with HRF: Ensuring EEG-fMRI Time Coherence

EEG signals provide millisecond-level temporal resolution, whereas fMRI data offer much lower temporal resolution owing to the sluggish nature of the hemodynamic response. Consequently, these modalities cannot be directly compared with their raw forms. Owing to this discrepancy, a direct comparison between the two modalities is not feasible. To compensate for this difference, EEG time series are convolved with the Hemodynamic Response Function (HRF), aligning them with the lower temporal resolution of the fMRI. HRF models the delayed and sluggish blood flow response to neural activity, thereby enhancing the temporal compatibility between EEG and fMRI data [2].

The HRF convolution is defined by the convolution integral:

(1) $$y\left(t\right)=\left(x*h\right)\left(t\right)=\int_{0}^{\infty }x(\tau )h(t-\tau )d\tau$$

$x\left(t\right)$ represents the EEG time series, $h\left(t\right)$ represents the canonical HRF and $y\left(t\right)$ represents the HRF-convolved EEG. This mathematical formula is standard in the literature [49,50,51] and ensures that HRF is a linear and deterministic process that does not produce spurious correlations.

#### 2.6.2. Correlation Analysis

Correlation analysis was performed to determine the functional relationships between EEG and fMRI signals. EEG-fMRI correlation maps were generated by calculating Pearson correlation coefficients (*rij*) between the HRF-convolved EEG time series and each voxel’s fMRI time series. Specifically, correlations were computed between the EEG spectral time series (300 × 32) and fMRI ICA time series (300 × 80) for each participant. This process was repeated separately for the five EEG frequency bands: delta, theta, alpha, beta, and gamma. The resulting correlation values produced functional connectivity maps, illustrating the spatial representation of the EEG frequency bands across the brain. In the generated brain graphs, specific brain regions (e.g., fMRI atlas regions or independent components) were defined as nodes and the correlation values between them were defined as edges. By modeling joint EEG-fMRI signals in a graph structure, this framework enables detailed examination of multimodal functional connections and provides insights into various brain networks.

#### 2.6.3. Static EEG-fMRI Brain Graphs

In static analysis, the correlation matrix (R) was assumed to remain constant over the entire recording period. This matrix was averaged over time to create static EEG-fMRI connectivity graphs. The size of the correlation matrix was *N* × *N* (*N* = 112; 32 EEG electrodes and 80 fMRI components). Two separate graphs were derived from this matrix: the positive correlation graph (*W*^+^), which includes only positive correlations, and the negative correlation graph (*W*^−^), which includes the absolute values of negative correlations. These static graphs represent time-averaged patterns of functional connectivity between the EEG and fMRI signals.

#### 2.6.4. Dynamic EEG-fMRI Brain Graphs

In the dynamic analysis, a sliding window approach was used to examine temporal fluctuations in brain connectivity, with a window size of 20 time points (TR) and a step size of 1 TR. For each window, the data were analyzed with a 1 TR shift, resulting in 281 windows (300 − 20 + 1 = 281). For each window, an individual correlation matrix was computed, and corresponding *W*^+^ and *W*^−^ graphs were generated. In total, 281 time-varying EEG-fMRI connectivity graphs were obtained for each subject.

These dynamic graphs reflect the temporal changes in multimodal synchronization, fluctuations in network topology, and evolving interactions between brain regions.

Together, static and dynamic brain graphs provide a comprehensive visualization of functional connectivity patterns over time, revealing the evolving nature of EEG-fMRI network integration.

#### 2.6.5. Brain Graph Evaluation Metrics

A network is a structure that mathematically models complex real-life systems. This structure consists of nodes and edges that represent the connections between nodes [52]. In this study, nodes represent brain regions, whereas edges represent connections between the two regions. Three fundamental network theory metrics—connection strength (CS), clustering coefficient (CC), and global efficiency (GE)—were computed using Brain Connectivity Toolbox (BCT) to evaluate both static and dynamic brain graphs [53]. CS measures the intensity of the interaction between two nodes [53]. In the context of a brain network, it represents the strength of the functional connectivity between two regions. The CS is calculated as the absolute value of the Pearson correlation between the signals:

(2) $${CS}_{ij}=\left|corr(x_{i},x_{j})\right|$$

where $x_{i}\;$ xi and $x_{j}$ represent the time series of the corresponding nodes. A higher CS value indicates stronger synchronization between the two regions. The CC indicates the density of connections between the neighbors of a node [54]. A high CC value suggests tightly connected local neighborhoods, reflecting modular structures in the brain. The CC for each node was calculated as follows:

(3) $${CC}_{i}=\frac{2E_{i}}{k_{i\;}(k_{i}-1)}$$

where $E_{i}$ is the number of edges between the neighbors of node i , and $k_{i\;}$ is the number of neighbors of node i. GE measures the efficiency of information transfer across an entire network [55]. It is defined as the average of the shortest path lengths between all the pairs of nodes.

(4) $$GE=\frac{1}{N(N-1)}\sum_{i\neq j}\frac{1}{d_{ij}}$$

where N is the total number of nodes and $d_{ij}$ is the shortest path length between nodes i and j. A higher GE value indicates faster and more efficient communication across networks. These three metrics were computed on both static and dynamic EEG-fMRI connectivity graphs to quantitatively evaluate the impact of artifact removal methods on brain network topology.

## 3. Results

### 3.1. Method Comparison on Signal Quality

**Figure 6:**
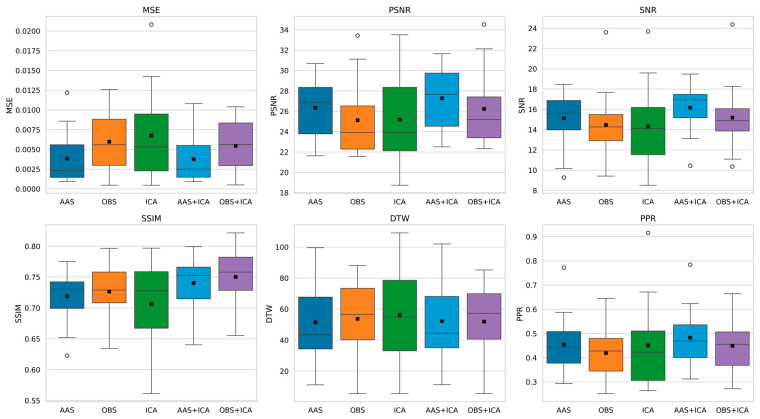
Boxplot comparison of artifact removal methods based on signal quality metrics (MSE, PSNR, SNR, SSIM, DTW, and PPR). Each boxplot summarizes the metric value distributions across participants for each method. The mean of the data distribution is shown as a black, bold multiplication sign, and the median is shown as a horizontal line within each box. White circular markers represent outliers.

**Figure 7:**
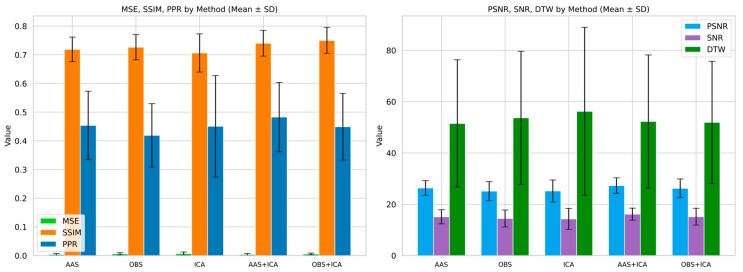
Barplot comparison of artifact removal methods based on average values of signal quality metrics (PSNR, SNR, DTW, MSE, SSIM, and PPR). Each bar shows the mean performance of a method, enabling comparisons across multiple quality dimensions.

The effectiveness of the artifact removal methods was evaluated using a range of quantitative metrics. Specifically, AAS, OBS, ICA, AAS + ICA, and OBS + ICA were compared based on metrics such as MSE, PSNR, SNR, SSIM, DTW, and PPR. This comparative analysis revealed the extent to which each method contributes to the signal quality and the effective removal of artifacts across multiple dimensions. Evaluating the different metrics allowed us to distinguish the relative strengths and limitations of each method more clearly. The performance results for these metrics are presented in detail in Figure 6 and Figure 7, respectively.

The analysis shows that the AAS method provides the closest result to the original signal with the lowest error rates and the highest signal quality in key metrics, such as MSE, PSNR, and SNR. These findings reveal that AAS minimizes signal degradation and effectively preserves the signal integrity. In addition, AAS achieved the best results in the DTW and PPR metrics, demonstrating its superiority in both temporal precision and amplitude structure preservation.

The OBS method showed the highest success, particularly in terms of the SSIM value, and stood out as the method that best preserved the structural features of the original signal. However, it performed less favorably than the AAS for the other metrics.

In general, ICA underperformed compared with AAS and OBS in terms of MSE, PSNR, SNR, and DTW metrics. However, it performed moderately in the SSIM and PPR metrics, indicating that it could partially preserve the structural and amplitude integrity of the signal. A detailed comparison of artifact removal methods based on signal quality metrics is provided in Appendix A (Table A1).

In general, ICA underperformed compared with AAS and OBS in terms of MSE, PSNR, SNR, and DTW metrics. However, it performed moderately in the SSIM and PPR metrics, indicating that it could partially preserve the structural and amplitude integrity of the signal.

The hybrid methods produced better overall results than the baseline methods.

- The AAS + ICA combination performed best in terms of MSE, PSNR, SNR, and PPR metrics, making it the most effective method in terms of both artifact removal success and signal quality.
- On the other hand, the OBS + ICA method gave the highest result in terms of SSIM value and best preserved the structural integrity of the signal.

**Figure 8:**
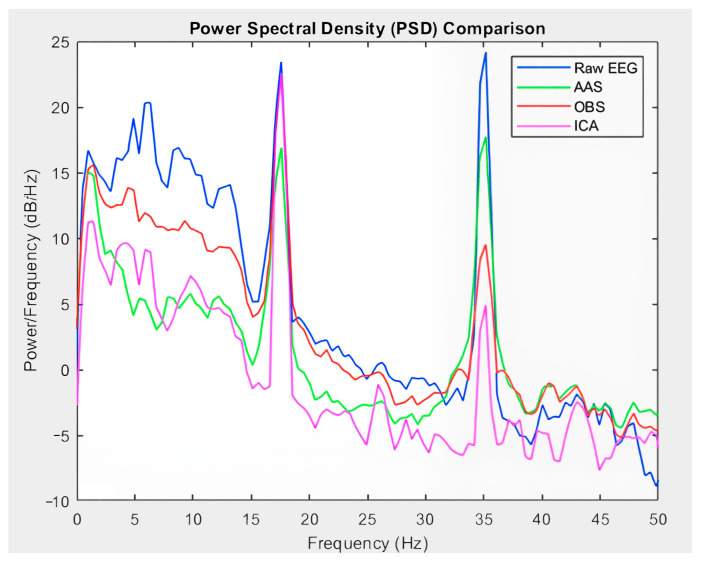
Comparison of PSD profiles between raw EEG signals and signals cleaned using different artifact removal methods (AAS, OBS, and ICA). The graph illustrates the spectral preservation across the methods.

These results suggest that hybrid approaches offer a more complete artifact removal performance, addressing the limitations of the individual methods in a complementary manner. The effects of the artifact removal methods on the frequency components of the EEG signal were evaluated using PSD analysis. Figure 8 illustrates the PSD profiles of the raw EEG signal and the signals cleaned using the AAS, OBS, and ICA methods, allowing a visual comparison of the spectral preservation across the methods.

The lower power levels in the PSD compared with the raw signal indicate that some frequency components are attenuated; however, the overall spectral structure is preserved, particularly within the critical frequency bands. Notably, the AAS and OBS methods better preserve the spectral power in the delta and alpha bands, whereas ICA exhibits a more aggressive filtering effect, resulting in the suppression of certain frequency components.

Computational efficiency differed markedly across the artifact removal strategies evaluated. As presented in Table 2, the standalone subtraction techniques exhibited substantially higher processing efficiency compared to the ICA-based methods. Among them, AAS achieved the shortest mean processing time of approximately 3 min per participant, followed by OBS at 4 min. In contrast, the ICA-based approaches incurred a considerably greater computational cost. Notably, the hybrid OBS + ICA method was the most time-intensive, requiring around 10 min per participant, reflecting the cumulative overhead introduced by its sequential multi-step processing pipeline.

### 3.2. Statistical Evaluation: Graph Metric Comparisons Across Frequency Bands

Dynamic graphs were computed using a 20-TR sliding window, consistent with prior EEG–fMRI studies [30,56,57]. To ensure robustness, additional analyses were performed using 10-TR and 30-TR windows.

In dynamic brain graphs, CS, CC, and GE, which are the basic graph metrics of brain networks, were calculated for different sliding window lengths and five EEG frequency bands (Delta, Theta, Alpha, Beta, Gamma), as summarized in Table 3. Overall, a decrease in CS, CC, and GE values is observed with increasing window length, indicating that shorter windows provide higher network transition sensitivity. While TR 10 shows the highest sensitivity, TR 20 offers a balance between capturing dynamic changes and ensuring statistical stability, making it the preferred window length for subsequent analyses.

**Figure 9:**
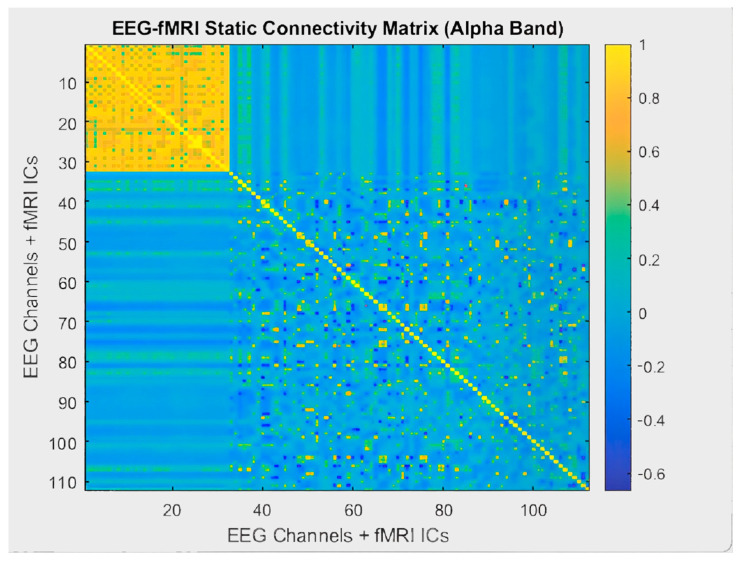
Static EEG-fMRI connectivity matrix for the alpha band. Yellow indicates positive correlations and blue indicates negative correlations, reflecting stable connectivity patterns between the EEG electrodes and fMRI components.

**Figure 10:**
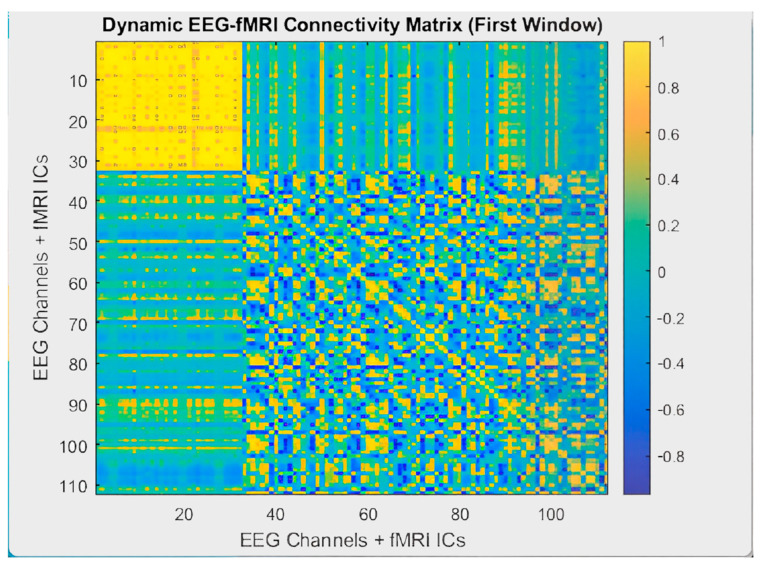
Dynamic EEG-fMRI connectivity matrix for the alpha band, computed using a sliding window (20 TR window, 1 TR step). Color variations reflect temporal changes in correlation strength and polarity, with yellow indicating high positive values and blue indicating negative values.

EEG-fMRI brain connectivity graphs were analyzed at static and dynamic levels. The static EEG-fMRI correlation matrix presented in Figure 9 was calculated by averaging correlations across the entire recording duration. This matrix represents the time-invariant functional connectivity patterns between brain regions. While strong correlations were observed between the EEG channels, a more spatially diffuse but fixed connectivity pattern was found among the fMRI components. In contrast, Figure 10 shows the dynamic EEG-fMRI connectivity matrix obtained using a sliding window approach. The yellow areas indicate strong positive correlations, whereas the blue areas indicate negative correlations. Compared to the static graph, the dynamic structure displayed greater variability and heterogeneity, highlighting temporal fluctuations in multimodal connectivity.

Static and dynamic brain graphs obtained using five different BCG artifact removal methods (AAS, OBS, ICA, AAS + ICA, and OBS + ICA) were statistically evaluated for significant differences across EEG frequency bands. For each method, paired *t*-tests were conducted to assess whether the graph metrics (CC, CS, and GE) differed among the frequency band pairs. To account for multiple comparisons across frequency band pairs and metrics, all *p*-values were corrected using the false discovery rate (FDR) method. The application of FDR correction mitigates the risk of false positives arising from multiple comparisons and ensures that the reported significance levels reflect robust statistical evidence. The results of these metrics are presented in Appendix B (Table A2, Table A3, Table A4, Table A5, Table A6, Table A7, Table A8, Table A9, Table A10 and Table A11), where statistical comparisons across frequency bands are reported.

**Figure 11:**
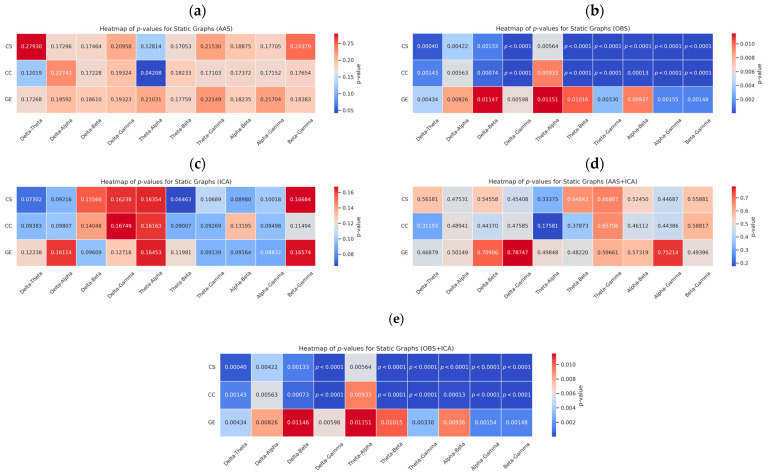
Heatmaps of *p*-values from static brain graphs obtained using the five BCG artifact removal methods: (**a**) AAS, (**b**) OBS, (**c**) ICA, (**d**) AAS + ICA, and (**e**) OBS + ICA. The maps illustrate differences (*p* < 0.05) in graph metrics (CS, CC, GE) between the EEG frequency band pairs. Blue indicates significance, red indicates non-significance, and color bars are scaled per method.

**Figure 12:**
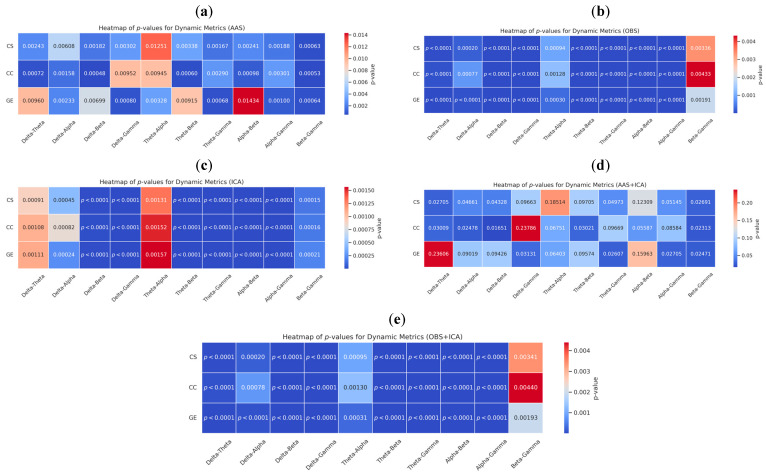
Heatmaps of *p*-values from dynamic brain graphs obtained using the five BCG artifact removal methods: (**a**) AAS, (**b**) OBS, (**c**) ICA, (**d**) AAS + ICA, and (**e**) OBS + ICA. The maps illustrate differences (*p* < 0.05) in graph metrics (CS, CC, GE) between the EEG frequency band pairs. Blue indicates significance, red indicates non-significance, and color bars are scaled per method.

This analysis aims to uncover the frequency-specific topological effects of artifact removal methods, not only at the signal level, but also at the network connectivity level. Figure 11 presents the *p*-value heatmaps from static graph comparisons, whereas Figure 12 shows the same for dynamic graphs. Each heatmap illustrates the extent to which the connectivity metrics (CS, CC, and GE) between different frequency bands were significantly altered. The OBS and OBS + ICA methods yielded statistically significant differences in most frequency band comparisons (*p* < 0.05, FDR-corrected), whereas AAS + ICA yielded fewer significant results, suggesting greater stability but lower discriminative power. These results indicate that artifact removal methods influence not only the overall connectivity strength, but also the frequency-dependent network organization. All *p*-values were derived using paired *t*-tests, with *p* < 0.05 considered statistically significant. The heat maps offer a visual summary of the *p*-values for each frequency band pair, with blue tones indicating significant differences, and red tones representing non-significant comparisons. Color gradients were scaled to enhance the visibility of the statistical significance levels.

The *p*-values obtained using the AAS method were generally high, indicating lower sensitivity in detecting statistical differences between the EEG frequency bands. Notably, no significant differences were observed among the delta, alpha, and gamma bands. In contrast, the OBS method yielded lower *p*-values, revealing statistically significant differences across frequency bands. In particular, low *p*-values in delta–theta, delta–alpha, theta–alpha, and theta–beta band pairs suggest the presence of distinct structural variations in these ranges. The ICA method also produced low *p*-values for most frequency band pairs, suggesting an enhanced ability to capture dynamic changes in EEG activity. Significant differences in the delta–gamma and alpha–beta pairs highlight ICA’s frequency-specific sensitivity of ICA. The consistently lower *p*-values obtained with the OBS and ICA methods suggest that these techniques are more sensitive to subtle variations in EEG-fMRI signals. In dynamic graph analyses, these methods produce stronger statistical differences, reflecting transient connectivity changes more effectively than static approaches.

Although the AAS method demonstrated limited sensitivity, particularly in static graphs, it performed better in some dynamic graph metrics. Moreover, combining AAS with ICA (AAS + ICA) resulted in more statistically significant differences than AAS alone. The already high performance of OBS was further enhanced when paired with ICA, and the OBS + ICA combination yielded the lowest *p*-values, particularly for the theta–beta, delta–gamma, and alpha–beta band pairs. A comparison of network graph metrics between static and dynamic brain graphs indicates that dynamic analyses produce more significant differences, as reflected by the lower *p*-values.

Among all methods, the OBS + ICA combination consistently yielded the lowest *p*-values in dynamic analyses, particularly in theta–beta, delta–gamma, and alpha–beta band pairs, indicating its superior sensitivity to transient connectivity changes. This result suggests that the complementary mechanisms of OBS and ICA enable more precise detection of short-term fluctuations in neural coupling, making this hybrid approach particularly effective for dynamic connectivity analysis.

This finding suggests that time-varying connectivity patterns capture details that remain invisible to static analysis. In particular, dynamic graphs revealed more significant differences in higher-frequency bands, such as beta and gamma, supporting their critical roles in cognitive and perceptual processes. The pronounced differences observed in the beta and gamma bands within dynamic analyses are consistent with the established roles of these frequency ranges in cognitive and perceptual functions. Beta-band oscillations (13–30 Hz) are typically associated with attentional control and sensorimotor processing, whereas gamma-band activity (30–80 Hz) reflects perceptual integration and working memory processes. Therefore, the enhanced distinction captured by OBS + ICA in these higher-frequency bands suggests improved preservation of functionally meaningful neural dynamics. Overall, these findings confirm that EEG-fMRI multimodal connectivity graphs provide a powerful framework for investigating brain function across both the temporal and spatial dimensions. Representing brain connectivity as nodes and edges within graph theory enables the systematic evaluation of interactions between regions. Exploring the topological manifestations of frequency-dependent functional differences offers novel insights into the organization of brain networks, especially in high-frequency components.

## 4. Discussion

Our study highlights several key findings regarding the effects of BCG artifact removal techniques on EEG signal quality and EEG-fMRI network connectivity. Specifically, AAS demonstrated the best overall signal quality, whereas OBS + ICA provided the strongest sensitivity to dynamic connectivity changes, particularly in high-frequency bands such as beta and gamma.

Although previous studies [10,11,13] have examined the effectiveness of these methods from certain perspectives, they have generally been evaluated using a limited number of metrics or one-way comparisons. In this study, different methods were analyzed using a holistic approach with multiple performance metrics and graph-theory-based connectivity analysis.

As expected, the AAS method had the lowest error and highest signal quality, particularly in metrics such as MSE, PSNR, and SNR. This shows that the method effectively removed artifacts while preserving the overall signal integrity to a large extent. However, OBS yielded higher values for the SSIM metric, suggesting that AAS is relatively limited in terms of preserving the structural properties of the signal. This finding may be due to the inability of the AAS to fully capture the temporal variability of artifacts, as suggested by Allen et al. [10].

In line with the literature, the OBS method showed the best performance in terms of preserving structural integrity. In particular, higher values of the SSIM metric indicate that the characteristic patterns of the signal are better preserved. However, it lags behind the AAS in terms of overall signal quality. Although this PCA-based approach improved the sensitivity to structural information, it did not achieve a balanced performance in all metrics.

Unexpectedly, although the ICA method exhibited lower signal quality metrics (MSE, SNR, and PSNR), it performed moderately well on structural similarity and timing coherence metrics, such as SSIM and PPR. This may be related to the specialized nature of component selection and the risk of information loss due to incorrect inferences.

In addition to the OBS + ICA approach proposed in the literature, the AAS + ICA method was evaluated in this study. Both hybrid structures provided significant performance improvements compared with the single methods. While AAS + ICA was particularly prominent in signal quality metrics (PSNR, SNR, and PPR), the OBS + ICA combination produced better results in terms of both structural integrity preservation and statistical significance. Another critical factor governing the choice of preprocessing pipeline is computational efficiency. Our analysis revealed a substantial application trade-off between methodological rigor and processing speed (Table 2). While the single-step subtraction methods were notably faster (AAS requiring 45 min and OBS 60 min per participant), the high computational demands of ICA resulted in significantly longer processing times. The most effective hybrid approach, OBS + ICA, was also the most time-consuming, requiring 240 min (4 h) per participant. This finding suggests that researchers conducting high-throughput studies or those with limited computational resources may prioritize the faster AAS or OBS methods. Conversely, for studies focused on highly sensitive analyses like dynamic functional connectivity, where the preservation of high-frequency band integrity is crucial, the substantial computational investment required by OBS + ICA is justified by its superior performance in revealing statistically significant dynamic link changes. Surprisingly, the high sensitivity of OBS + ICA with low *p*-values makes it a strong candidate for a dynamic link analysis.

One of the important contributions of this study is the evaluation of the topological effects of different artifact removal methods on signal quality as well as EEG-fMRI brain graphs. Analyses with metrics such as CS, CC, and GE showed that each method produced different effects on network topology.

In the static plots, AAS produced less significant differences (high *p*-values), whereas OBS and ICA showed more significant changes across the frequency bands. In the dynamic plots, all methods exhibited lower *p*-values, indicating that they were able to capture the evolution of links over time more precisely. This finding is in line with that of [30], who demonstrated that dynamic link analysis provides a more sensitive assessment than static methods.

A particularly striking result was that high-frequency bands (beta and gamma) showed more significant differences in the dynamic connectivity analyses. Beta band activity is strongly implicated in maintaining the current sensorimotor state and motor inhibition, and its interaction with fMRI signals often reflects sustained attention and memory encoding. Similarly, gamma band oscillations are known to support higher-order cognitive processes, including working memory and perceptual binding. The observation that high-frequency bands (e.g., beta and gamma) exhibit the most pronounced differences under dynamic conditions is particularly insightful, as these bands are critically involved in complex, time-locked cognitive functions. Beta band activity (typically 13–30 Hz) is strongly implicated in maintaining the current sensorimotor state and motor inhibition, while gamma band oscillations (typically >30 Hz) are known to support higher-order cognitive processes, including working memory and perceptual binding. The fact that OBS + ICA significantly enhances the resolution of these bands in dynamic connectivity suggests that residual artifacts severely contaminate these crucial, yet weaker, high-frequency neural signals. Therefore, methodological choices directly determine the viability of extracting meaningful connectivity patterns related to high-level cognition. This finding supports the critical role of these bands in cognitive and perceptual processes. The OBS + ICA combination revealed strong differences in frequency pairs, such as theta and delta gamma, suggesting that it is a preferable method for analysis in these areas. The superior performance of the OBS + ICA combination in dynamic analyses can be attributed to the complementary functioning of both algorithms. While OBS effectively reduces gradient and pulse-related artifacts through principal component-based filtering, ICA subsequently separates and eliminates residual independent artifact sources. This sequential process preserves the temporal fidelity of the EEG signal and enhances its sensitivity to fast-varying connectivity changes, providing a methodological advantage for dynamic functional analysis.

A potential limitation of this study is that the dataset includes only 20 male participants, which may constrain the generalizability of our findings. Future studies should include larger and more diverse samples to evaluate the robustness of BCG artifact removal methods across sexes, age ranges, and clinical populations. Another limitation of the present study is the lack of external validation using an independent dataset. Although consistent results were obtained across multiple signal quality and graph-theoretical metrics, future research should replicate these findings using different EEG-fMRI datasets to confirm their generalizability. Employing cross-dataset validation or leave-one-subject-out procedures could further strengthen the robustness of the observed methodological differences.

Finally, although this study focused solely on resting-state EEG-fMRI data to maintain experimental control and minimize task-related variability, future research could extend the analysis to task-based conditions. Evaluating BCG artifact removal performance during cognitively demanding tasks would provide valuable insights into the robustness and generalizability of the compared methods under dynamic neural states.

## 5. Conclusions

This study provides a comprehensive evaluation of three widely used methods for the removal of BCG artifacts from simultaneous EEG-fMRI recordings: AAS, OBS, and ICA. Our findings demonstrate that each method has distinct strengths: AAS ensures superior signal quality; OBS excels in preserving structural features; and ICA offers moderate performance across both dimensions. Hybrid methods, particularly OBS + ICA, have shown promising results in dynamic connectivity analyses, highlighting their potential for future multimodal studies.

This is the first study to compare five BCG removal pipelines in both static and dynamic brain graph contexts. The integration of graph-theoretical analyses allowed us to explore the topological effects of artifact correction methods on brain networks, revealing that dynamic approaches are more sensitive than static evaluations, particularly in high-frequency bands. Unlike previous studies that focused on either EEG or fMRI metrics, we integrated graph metrics to evaluate the multimodal integrity. In previous studies, the use of combined methods (OBS + ICA) and graph-based metrics for evaluation has been underexplored.

Despite its strengths, this study had some limitations. Limitations of the study include the fact that the dataset used belonged to a specific age and demographic group. In addition, these methods have only been applied to resting-state EEG-fMRI data. In future studies, it will be important to apply the same methodology to larger populations and task-based paradigms to evaluate the generalizability of the method.

## Figures and Tables

**Table 1:** Metrics Used to Evaluate the Impact of Artifact Removal Methods on Signal Quality.

Metrics	Description	Optimal Direction	Mathematical Expression
MSE	Mean squared difference between the original and processed signal [37]	Low	$MSE=\frac{1}{n}\sum_{i=0}^{n}{{(x}_{i}-y_{i})}^{2}$ $x_{i}$: Original signal, $y_{i}$: Filtered signal n: Total number of samples
PSNR	Amount of distortion in the signal [38]	High	$PSNR={10\log}_{10}\left(\frac{{MAX}^{2}}{MSE}\right)$ MAX: Maximum possible value of the signal
SNR	Ratio of signal power to noise power [39]	High	$SNR={10\log}_{10}\left(\frac{{P_{signal}}^{2}}{P_{noise}}\right)$ $P_{signal}$: Power of the original signal $P_{noise}$: Power of the noise
SSIM	Structural similarity between signals [40]	High	$SSIM\;\left(x,y\right)=\frac{\left(2\mu_{x}\mu_{y}+C_{1})(2\sigma_{xy}+C_{2}\right)}{\left({\mu_{x}}^{2}+{\mu_{y}}^{2}+C_{1})({\sigma_{x}}^{2}+{\sigma_{y}}^{2}+C_{2}\right)}$ $\mu_{x}{,\;\mu }_{y}$: Mean of original and filtered signals, ${\sigma_{x}}^{2},\;{\sigma_{y}}^{2}$: Variance of original and filtered signals $\sigma_{xy}$: Covariance between the signals $C_{1}=\;{\left(k_{1}L\right)}^{2}$ and $C_{2}=\;{\left(k_{2}L\right)}^{2}$ and $k_{1\;}=0.01,\;k_{2\;}=0.03\;and\;L=2^{\#bits\;per\;pixel}-1$
DTW	Temporal similarity between signals [41]	Low	$DTW\;\left(x,y\right)=min\sum_{i=0}^{n}d(x_{i},\;y_{i})$ x, y: Two time series $d(x_{i},\;y_{i})$: Distance between corresponding elements
PPR	Ratio of amplitude change [13]	Close to 1	$PTPR=\;\frac{\max\left(y\right)-min(y)}{\max\left(x\right)-min(x)}$ max(y), min(y): Maximum and minimum of the filtered signal max(x), min(x): Maximum and minimum of the original signal
PSD	Conservation of frequency components [42]	Spectral Integrity	$PSD\;\left(f\right)=\underset{n\to \infty }{\lim}\frac{1}{T}\;E\left[{\left|X(f)\right|}^{2}\right]$ X(f): Fourier transform of the signal T: Total duration of the signal.

**Table 2:** Average processing time per participant and total processing time for all participants for each BCG trace extraction method.

Methods	Average Processing Time	Total Processing Time
AAS	3 min	45 min
OBS	4 min	60 min
ICA	10 min	150 min
AAS + ICA	15 min	225 min
OBS + ICA	16 min	240 min

**Table 3:** Mean ± Standard Deviation values of dynamic brain networks metrics (CS, CC, GE) for different sliding window lengths (TR 10, 20, 30) and EEG frequency bands (Delta, Theta, Alpha, Beta, Gamma).

Metric	TR	Delta	Theta	Alpha	Beta	Gamma
CS	10	30.368 ± 0.773	30.444 ± 0.868	30.746 ± 0.804	29.436 ± 0.851	28.865 ± 0.586
	20	23.280 ± 0.881	23.502 ± 0.882	23.718 ± 0.834	22.250 ± 0.994	21.595 ± 0.685
	30	20.207 ± 0.962	20.430 ± 0.879	20.608 ± 0.834	19.126 ± 1.110	18.345 ± 0.738
CC	10	0.428 ± 0.011	0.428 ± 0.012	0.432 ± 0.011	0.419 ± 0.010	0.414 ± 0.010
	20	0.314 ± 0.013	0.316 ± 0.013	0.319 ± 0.012	0.302 ± 0.013	0.295 ± 0.010
	30	0.272 ± 0.016	0.275 ± 0.015	0.276 ± 0.013	0.259 ± 0.015	0.249 ± 0.011
GE	10	0.734 ± 0.003	0.735 ± 0.003	0.736 ± 0.003	0.729 ± 0.003	0.727 ± 0.002
	20	0.726 ± 0.003	0.727 ± 0.003	0.728 ± 0.003	0.720 ± 0.004	0.717 ± 0.003
	30	0.718 ± 0.004	0.719 ± 0.004	0.720 ± 0.003	0.712 ± 0.005	0.708 ± 0.003

## Data Availability

The datasets used and analyzed during the current study are available in the Mendeley Data Repository, accessed on 20 August 2024 [https://data.mendeley.com/datasets/crhybxpdy6/1].

## Abbreviations

The following abbreviations are used in this manuscript:

AAS	Average Artifact Subtraction
BCG	Directory of open access journals
BIDS	Brain imaging data structure
CC	Clustering coefficient
CS	Connection strength
DTW	Dynamic time warping
DMN	Default mode network
EC	Eyes closed
EEG	Electroencephalography
EPI	Echo planar imaging
EO	Eyes open
FDR	False discovery rate
FOV	Field of view
FFT	Fast Fourier transform
fMRI	Functional magnetic resonance imaging
GE	Global efficiency
GIFT	Group ICA of the fMRI toolbox
HRF	Hemodynamic response function
ICA	Independent component analysis
ICN	Independent component network
IQ	Stability index
JICA	Joint independent component analysis
MSE	Mean squared error
OBS	Optimal basis set
PCA	Principal component analysis
PSD	Power spectral density
PPR	Peak-to-peak ratio
SNR	Signal-to-noise ratio
SSIM	Structural similarity index
TR	Repetition time
TE	Echo time

## Appendix A

**Table A1:** Comparison of artifact removal methods according to signal quality metrics.

Mean ± SD	MSE	PSNR (dB)	SNR (dB)	SSIM	DTW	PPR
AAS	0.0038 ± 0.0032	26.3484 ± 2.8016	15.1198 ± 2.6563	0.7188 ± 0.0413	51.4970 ± 23.9658	0.4538 ± 0.1152
OBS	0.0059 ± 0.0036	25.1214 ± 3.5812	14.4701 ± 3.1858	0.7264 ± 0.0425	53.7106 ± 25.0494	0.4190 ± 0.1067
ICA	0.0067 ± 0.0043	25.1823 ± 4.0892	14.3125 ± 3.8654	0.7063 ± 0.0641	56.2264 ± 29.5970	0.4507 ± 0.1684
AAS + ICA	0.0035 ± 0.0031	27.2753 ± 2.9333	16.1648 ± 2.2601	0.7403 ± 0.0433	52.2920 ± 25.0441	0.4828 ± 0.1167
OBS + ICA	0.0054 ± 0.0031	26.2289 ± 3.5166	15.1749 ± 3.1685	0.7502 ± 0.0440	51.9202 ± 22.9925	0.4491 ± 0.1123

## Appendix B

**Table A2:** *p*-values for static graphs obtained as a result of AAS method.

Frequency Band Pairs	CS	CC	GE
Delta-Theta	0.27930	0.12019	0.17268
Delta-Alpha	0.17296	0.22743	0.19592
Delta-Beta	0.17464	0.17228	0.18610
Delta-Gamma	0.20958	0.19324	0.19323
Theta-Alpha	0.12814	0.04208	0.21031
Theta-Beta	0.17053	0.18233	0.17759
Theta-Gamma	0.21530	0.17103	0.22149
Alpha-Beta	0.18875	0.17372	0.18235
Alpha-Gamma	0.17705	0.17152	0.21704
Beta-Gamma	0.24379	0.17654	0.18383

**Table A3:** *p*-values for static graphs obtained as a result of OBS method.

Frequency Band Pairs	CS	CC	GE
Delta-Theta	0.0004	0.0014	0.0043
Delta-Alpha	0.0042	0.0056	0.0082
Delta-Beta	0.0013	0.0007	0.0114
Delta-Gamma	0.00001	0.000005	0.0059
Theta-Alpha	0.0056	0.0093	0.0115
Theta-Beta	0.000006	0.000005	0.01015
Theta-Gamma	0.0000002	0.00000006	0.0033
Alpha-Beta	0.00006	0.0001	0.0093
Alpha-Gamma	0.00000006	0.0000003	0.0015
Beta-Gamma	0.00001	0.00001	0.0014

**Table A4:** *p*-values for static graphs obtained as a result of ICA method.

Frequency Band Pairs	CS	CC	GE
Delta-Theta	0.0730	0.0938	0.1223
Delta-Alpha	0.0921	0.0980	0.1611
Delta-Beta	0.1556	0.1404	0.0960
Delta-Gamma	0.1623	0.1674	0.1271
Theta-Alpha	0.1635	0.1616	0.1645
Theta-Beta	0.0646	0.0900	0.1198
Theta-Gamma	0.1068	0.0926	0.0913
Alpha-Beta	0.0897	0.1319	0.0916
Alpha-Gamma	0.1001	0.0949	0.0883
Beta-Gamma	0.1668	0.1149	0.1657

**Table A5:** *p*-values for static graphs obtained as a result of AAS + ICA method.

Frequency Band Pairs	CS	CC	GE
Delta-Theta	0.5618	0.3119	0.4687
Delta-Alpha	0.4753	0.4894	0.5014
Delta-Beta	0.5455	0.4437	0.7090
Delta-Gamma	0.4540	0.4758	0.7874
Theta-Alpha	0.3337	0.1758	0.4984
Theta-Beta	0.6484	0.3787	0.4822
Theta-Gamma	0.6688	0.6570	0.5966
Alpha-Beta	0.5245	0.4611	0.5731
Alpha-Gamma	0.4468	0.4438	0.7521
Beta-Gamma	0.5588	0.5881	0.4939

**Table A6:** *p*-values for static graphs obtained as a result of OBS + ICA method.

Frequency Band Pairs	CS	CC	GE
Delta-Theta	0.0004	0.0014	0.0043
Delta-Alpha	0.0042	0.0056	0.0082
Delta-Beta	0.0013	0.0007	0.0114
Delta-Gamma	0.00001	0.000005	0.0059
Theta-Alpha	0.0056	0.0093	0.0115
Theta-Beta	0.000006	0.000005	0.0101
Theta-Gamma	0.0000002	0.0000006	0.0033
Alpha-Beta	0.00006	0.0001	0.0093
Alpha-Gamma	0.00000006	0.00000003	0.0015
Beta-Gamma	0.00001	0.00001	0.0014

**Table A7:** *p*-values for dynamic graphs obtained as a result of AAS method.

Frequency Band Pairs	CS	CC	GE
Delta-Theta	0.0024	0.0007	0.0095
Delta-Alpha	0.0060	0.0015	0.0023
Delta-Beta	0.0018	0.0004	0.0069
Delta-Gamma	0.0030	0.0095	0.0008
Theta-Alpha	0.0125	0.0094	0.0032
Theta-Beta	0.0033	0.0005	0.0091
Theta-Gamma	0.0016	0.0028	0.0006
Alpha-Beta	0.0024	0.0009	0.0143
Alpha-Gamma	0.0018	0.0030	0.0009
Beta-Gamma	0.0006	0.0005	0.0006

**Table A8:** *p*-values for dynamic graphs obtained as a result of OBS method.

Frequency Band Pairs	CS	CC	GE
Delta-Theta	0.00001	0.00008	0.00001
Delta-Alpha	0.00019	0.00077	0.00007
Delta-Beta	0.0000003	0.00000005	0.00001
Delta-Gamma	0.000004	0.000007	0.000003
Theta-Alpha	0.00093	0.00127	0.0003
Theta-Beta	0.0000006	0.0000001	0.000001
Theta-Gamma	0.0000007	0.000001	0.0000003
Alpha-Beta	0.0000001	0.00000007	0.0000004
Alpha-Gamma	0.000003	0.000006	0.000001
Beta-Gamma	0.0033	0.0043	0.0019

**Table A9:** *p*-values for dynamic graphs obtained as a result of ICA method.

Frequency Band Pairs	CS	CC	GE
Delta-Theta	0.0009	0.00108	0.00111
Delta-Alpha	0.0004	0.0008	0.00023
Delta-Beta	0.000007	0.00001	0.000007
Delta-Gamma	0.00000005	0.0000001	0.00000006
Theta-Alpha	0.0013	0.0015	0.0015
Theta-Beta	0.0000007	0.0000007	0.000003
Theta-Gamma	0.00000003	0.00000005	0.00000005
Alpha-Beta	0.00000004	0.00000004	0.00000009
Alpha-Gamma	0.00000001	0.00000009	0.00000008
Beta-Gamma	0.000152	0.000159	0.0002

**Table A10:** *p*-values for dynamic graphs obtained as a result of AAS + ICA method.

Frequency Band Pairs	CS	CC	GE
Delta-Theta	0.0270	0.0300	0.2360
Delta-Alpha	0.0466	0.0247	0.0901
Delta-Beta	0.0432	0.0165	0.0942
Delta-Gamma	0.0966	0.2378	0.0313
Theta-Alpha	0.1851	0.0675	0.0640
Theta-Beta	0.0970	0.0302	0.0957
Theta-Gamma	0.0497	0.0966	0.0260
Alpha-Beta	0.1230	0.0558	0.1596
Alpha-Gamma	0.0514	0.0858	0.0270
Beta-Gamma	0.0269	0.0231	0.0247

**Table A11:** *p*-values for dynamic graphs obtained as a result of OBS + ICA method.

Frequency Band Pairs	CS	CC	GE
Delta-Theta	0.00001	0.00008	0.00001
Delta-Alpha	0.00019	0.00078	0.00007
Delta-Beta	0.0000003	0.0000005	0.00001
Delta-Gamma	0.000004	0.000007	0.000003
Theta-Alpha	0.0009	0.0012	0.0003
Theta-Beta	0.00000006	0.00000001	0.000001
Theta-Gamma	0.0000008	0.000001	0.0000003
Alpha-Beta	0.00000001	0.00000006	0.0000004
Alpha-Gamma	0.000003	0.000006	0.000001
Beta-Gamma	0.0034	0.0044	0.0019
